# β-cyclodextrin cross-linked metal organic frameworks as a new sensing candidate for donepezil hydrochloride potentiometric sensors

**DOI:** 10.1186/s13065-025-01521-2

**Published:** 2025-05-29

**Authors:** Rehab O. El-Attar, Reda M. Abdelhameed, Elmorsy Khaled

**Affiliations:** 1 Microanalysis Laboratory, Applied Organic Chemistry Department, National Research Centre, El Bohouth St., Dokki, Giza, 12622 Egypt; 2 Applied Organic Chemistry Department, National Research Center, El Bohouth St., Dokki, Giza, 12622 Egypt

**Keywords:** Donepezil hydrochloride, Planner disposable sensors, β-cyclodextrin cross-linked metal–organic frameworks, Pharmaceutical analysis, Biological samples, Dissolution studies

## Abstract

Screen-printing is a well-established promising technology for large scale production of planner disposable electrochemical sensors. The present study aims to fabricate a novel donepezil hydrochloride (DPH) screen-printed sensor integrated with the cross-linked β-cyclodextrin-functionalized aluminum metal organic framework-multiwall carbon nanotubes nanocomposites (β-CD/MOF/MWCNTs) as a novel sensing element. The fabricated disposable sensors exhibit theoretical Nernstian compliance value of 60.7 ± 1.5 mV decade^−1^ within a linear dynamic concentration range from 10^–6^ to 10^–2^ mol L^−1^ and limit of detection 7.0 × 10 ^−7^ molL^−1^. The DPH disposable sensors show high potential stability with a prolonged operational lifetime and the fast response time of 6 s. The presented electrochemical sensors represent an efficient analytical tool for fast and sensitive assay of DPH residues in the marketed pharmaceutical tablets and biological samples with acceptable average recoveries under direct potentiometric measurements, flow injection analysis (FIA), and potentiometric titration. Moreover, the dissolution and degradation studies of DPH can be monitored by the presented disposable sensors.

**Supplementary Information:**

The online version contains supplementary material available at 10.1186/s13065-025-01521-2.

## Introduction

Based on their high surface/volume ratio, nanomaterials brought challenging and promising opportunities for developing the analytical performance of electrochemical sensors [1–6]. Recently, the progress in the synthesis of crystalline porous materials found tremendous attention, where metal–organic framework (MOF) represents a new member in the vast field of porous three-dimensional coordination materials combining the advantages of both organic and inorganic materials [7, 8]. The organic linkers, central metal ions, and sequentially structural motifs yield infinite possibilities of MOF structures and combinations [9–12]. The functionalized MOFs can be cross-linked with various macromolecules with tunable properties and the synergic effect of both components. Post synthesis modification of MOFs with cyclodextrins (CDs) and other macrocyclic compounds assures synergistic performance of both the nanostructure of MOFs and the inclusion complex formation of CDs with the target analyte [13–19]. Therefore, MOFs regarded as promising candidates for drug delivery, gas sorption, ion exchange, photocatalysis, separation, water and energy harvesting [8, 10, 11, 20]. Also, combination of functionalized MOFs with different carbonaceous nanostructures creates effective sensing candidates for integrated electrochemical sensors [21–25].

Donepezil hydrochloride (DPH, ± -2-[(1-Benzyl-4-piperidyl) methyl]-5,6-dimethoxy-1-indanone hydrochloride), is a reversible acetylcholinesterase enzyme (AChE) inhibitor which enhances the acetylcholine availability at the synapses with good communication among nerve cells and promoted cholinergic function [26, 27]. DPH has been administrated Alzheimer's dementia symptomatic treatment [27].

The official pharmacopoeial analytical approach for DPH based on chromatographic techniques [28]. However, different analytical approaches and research articles have been developed for measuring the DPH in biological fluids and pharmaceutical formulations [29]. Chromatographic techniques including HPLC–MS [30], HPLC–UV [31, 32], and diode-array (HPLC–DAD) [33] were the most common. Nerveless, spectrofluorimetric and spectrophotometric measurements were also reported as an alternative choice for routine analysis of DPH in pharmaceutical forms [34–37]. Even though the aforementioned analytical approaches offer the required sensitivity and selectivity towards DPH; they are time-consuming with tedious analysis protocol and operate with high-cost instrumentations which obstacle their applications for high sample frequency and field monitoring of pharmaceutical residues in environmental samples. As a common and popular Alzheimer's medicine; developing of a new convenient and simple analytical method for determination of DPH residues is welcomed.

Electroanalytical measurements based on tailor-made electrochemical sensors possess the advantages of fast nondestructive measurement with low-cost equipment, [38]. Potentiometric sensors are considered as the most common and simple electrochemical sensors for monitoring of pharmaceutical residues [39–41]. The reported DPH potentiometric sensors were based on PVC membrane, solid-state, or carbon paste electrodes incorporated with either tetraphenylborate-DPH ion associates or cyclodextrin as electroactive materials [42–44]. However, these electrodes showed inherent limitations as they are too bulky with limited operational lifetimes and the requirement of sterilization for biomedical applications. As the consumption of pharmaceutical compounds continues to rise, disposable commercial sensors are adopted as a part of portable devices from “lab-to-market. Screen printing methodology is a common approach for commercialization of planner electrochemical sensors with high manufacturing reproducibility [45–49].

Molecular recognition through formation of inclusion complexes introduced alternative approaches for enhancing the performance of the electrochemical sensors regarding their sensitivity and selectivity [50–53]. The effective attractive host–guest interactions forces included van der Waals forces, π–π interaction, dipole–dipole, or hydrogen bonding. Among various macrocyclic compounds, cyclodextrins (CDs) were reported as the most popular sensing ionophores for electrochemical sensors. With a lipophobic interior cavity, CDs offer a suitable environment for fitting the gust molecule. The stability constants of the formed inclusion complexes are governed by the CD cavity size compatible with the guest molecule and the side substitution of the CD ring.

The present study aims to introduce cyclodextrins and other macrocyclic compounds including crown ethers, calixarenes, and their nanocomposites with metal organic frameworks as sensing element for potentiometric determination of DPH in the marketed pharmaceutical tablets and biological samples. Moreover, the presented sensors were introduced for monitoring the dissolution and degradation studies of DPH.

## Experimental

### Reagents and chemicals

All chemicals were analytical grade and bidistilled water (Milli-Q system, Millipore) was used in the present study. Different macrocyclic compounds, (supplied by Sigma-Aldrich), were tested as electroactive sensing elements including; α-CD (**I**), γ-CD (**II**), β-CD (**III**), 2, 6-di-O-methyl-β-CD (**IV**), 2, 3, 6-tri-O-methyl-β-CD (**V**), 12-crown-4 ether (**VI**), 15-crown-5 ether (**VII**), 18-crown-6 ether (**VIII**), 21-crown-7 ether (**IX**), dibenzo-24-crown-8 ether (**X**), 30-crown-10 ether (**XI**), calix-4-arene (**XI**I), and calix-8-arene (**XIII**).

The following tetraphenylborate derivatives were used as lipophilic additives in the sensing membrane, sodium tetraphenylborate (NaTPB, Fluka), potassium tetrakis (4-chlorophenyl) borate (KTClPB, Fluka), and sodium tetrakis (4-fluorophenyl) borate (NaTFPB, Fluka). Tricresylphosphate (TCP, Fluka), dioctylsebacate (DOS, Avocado), dioctylphthalate (DOP, Sigma), *o*-nitro-phenyloctylether (*o*-NPOE, Sigma), and 2-fluorophenyl-2-nitrophenyl ether (*f*-PNPE, Fluka) were used as plasticizers.

Graphite powder and high molecular weight polyvinyl chloride (supplied by Aldrich) were applied for formulation of the homemade printing carbon ink and the sensing membrane matrix. Carbon nanotubes, either multiwall carbon nanotubes (MWCNTs, Aldrich) or single wall carbon nanotubes (SWCNTs, Aldrich) were used for synthesis of the nanocomposite with the cross-linked CD-MOFs complex.

### Authentic donepezil sample and the stock solution

Donepezil hydrochloride (DPH, C_24_H_29_ClNO_3_, 379.49 gmol^–1^) standard sample was obtained from the Arab Drug Company (ADCo, Cairo, Egypt). The stock drug solution (10^–2^ molL^−1^) was prepared by dissolving a calculated amount of DPH authentic sample in water and kept in regenerator at 4 °C. Other DPH working solutions within the concentrations range from 1 × 10^–3^ to 1 × 10^–7^ mol L^−1^ were prepared by the serial dilution of the stock DPH solution.

### DPH pharmaceutical and biological samples

Donepezil pharmaceutical formulations (Aricept; 5 mg DPH/tablet; ADCo) were obtained from local markets. Five tablets were crushed to fine powder, dissolved in 50 mL water, and sonicated for 15 min. The DPH solution was filtrated and the drug content was assayed according to the pharmacopoeial protocol and the presented potentiometric sensor.

The standard plasma samples (purchased from VACSERA, Giza, Egypt) were enriched with known increments of the DPH stock solution, and mixed with acetonitrile (3:1 ratio). The fortified sample was centrifuged for 10 min to remove the residual sample protein. Urine sample were enriched with known aliquots of the stock DPH solution and mixed with equal volumes of methanol. The DPH contents in the clear supernatants were analyzed potentiometrically and according to the pharmacopoeial protocol.

### Measuring system

A portable Digital Multimeter with a PC interface (Radioshack, China) and 692-pH meter with a combined glass electrode (Metrohm, Switzerland) were used for potentiometric measurements and pH measurements, respectively. A single line flow injection system composed of four channels peristaltic pump (MCP Ismatec, Zurich, Switzerland), sample injection valve (ECOM, VentilC, Czech Republic), and home-designed continuous flow cells [54] was applied for flow injection analysis (FIA).

### Synthesis of metal organic framework- cross-linked macromolecule composite

#### *Synthesis of Al MOF (MIL-53-NH*_*2*_*)*

The amino functionalized MIL-53(Al) metal organic framework was synthesized and characterized as described in details elsewhere [17]. Briefly, the teflonlined stainless steel bomb filled with 28 mL water containing 0.120 g (0.66 mmol) of 2-amino-benzenedicarboxylic acid and 0.56 mL (0.22 mmol) of NaOH solutions were mixed with 1.10 mL (0.44 mmol) AlCl_3_.6H_2_O solution. The reaction mixture was heated at 110 °C for 24 h to complete the crystal growth. The formed MOF precipitate was washed several times with distilled water, DMF, soaked for 24 h in CH_2_Cl_2_, and dried at 80 °C.

##### *Preparation of MIL-53-NH*_*2*_*- cross-linked cyclodextrin composite*

Two grams of the native β-CD was dissolved in 100 mL of acetic acid (2%) and mixed with 0.5 mL of epichlorohydrine at room temperature. The reaction mixture was kept at 60 °C for 4 h, incubated overnight at 50 °C, and the obtained solids were washed carefully to obtain pure cross linked cyclodextrin derivative.

Cyclodextrin-MOF composite was designed via post synthetic modification technique (Figure S1). The fresh cross-linked cyclodextrin was mixed with MIL-53-NH_2_ in ultrasonic bath for about 2 h. The resultant solution was completed with another two hours in sonicated water bath keeping the MIL-53-NH_2_ ratio at 20% from the total solid cyclodextrin content.

##### *Synthesis of MIL-53-NH*_*2*_*-cross-linked crown ether and calixarene composites*

Selected other macromolecules, 18-crown-6 ether (**VIII**), and calix-8-arene (**XIII**), were cross-linked with the functionalized MIL-53(Al)-NH_2_ as described in details elsewhere [17, 18].

### Construction of the disposable DPH sensors

The potentiometric-screen printed sensors were deposited on the PVC sheet using graphite and silver-silver chloride printing inks as described elsewhere [55]. The sensing membrane matrix was formulated by dissolving 2.0 mg of the MIL-53(Al)-NH_2_-β-CD nanocomposite and 1.0 mg NaTFPB in 360 mg *f*-PNPE with continues stirring for 15 min. Next, 6 mL tetrahydrofuran, 240 mg PVC, and 15.0 mg MWCNTs were added and the sensing cocktail sonicated for 2 h. 20 µL of the cocktail were drop-casted on the printed graphite/PVC track and left to dry for 24 h at 25 °C. The fresh fabricated sensors were preconditioned in 10^–3^ molL^−1^ DPH solution for 5 min before using.

### Measuring procedures

The constructed sensors were immersed in the measuring cell containing DPH solution within the ascending concentrations ranged from 10^–7^ to 10^–2^ mol L^−1^ at room temperature. The steady state potential values were plotted against the corresponding DPH concentration in logarithmic scale [56]. For FIA, 50 μL of the authentic DPH solutions were injected in the carrier solution at the flow rate of 12.6 mL min^−1^ and the electrode potential was monitored against time. The recorded peak heights were plotted against the corresponding DPH concentration [54].

### Analysis of samples

The contents of DPH in the marketed pharmaceutical formulation and biological fluids were assayed using the developed DPH disposable potentiometric sensors in comparison with the official procedures. Following the standard addition protocol, known aliquots of the stock DPH solution were mixed with the sample solution. For each increment, the steady state electrode potentials were measured and the DPH concentration in the sample solution was estimated [57].

For FIA measuring, 50 µL of the DPH sample solution were injected in the carrier stream and the recorded peak height was compared with those recorded for injection of the standard DPH solutions of the same concentration.

The DPH samples containing 0.379 to 1.895 mg were potentiometrically titrated against the standardized NaTPB solution using the β-CD/MOF/MWCNTs/SPE as indicator electrode. After each addition, the steady state potential readings were recorded and plotted versus NaTPB volume. The end point was estimated from the first derivative of the sigmoid-shape titration curves [58].

### Donepezil tablet dissolution profile

One pharmaceutical tablet was put in the dissolution vessel filled with 50 mL of 0.1 mol HCl solution at 37 °C with a rotation speed 50 rpm [59]. After selected time intervals, 4 mL of the sample solution were withdrawn from the dissolution solution. The released DPH amounts in the dissolution medium were estimated spectrophotometrically at 271 nm and potentiometrically with the introduced DPH sensor [59, 60].

### Forced degradation studies of DPH

Different stress degradation protocols were applied to the authentic DPH sample. The stock DPH solutions (100 µg mL^−1^), was undergone to thermal stress (70 °C), alkaline stress in NaOH solution, acidic hydrolysis in HCl solution, light exposure, and oxidative stress using H_2_O_2_ solution at room temperature for seven days. The degradation process was followed using spectrophotometer at 271 nm at different time intervals [61].

## Results and discussion

### Characterization of the β-CD- cross linked metal organic frameworks composite

**Fig. 1:**
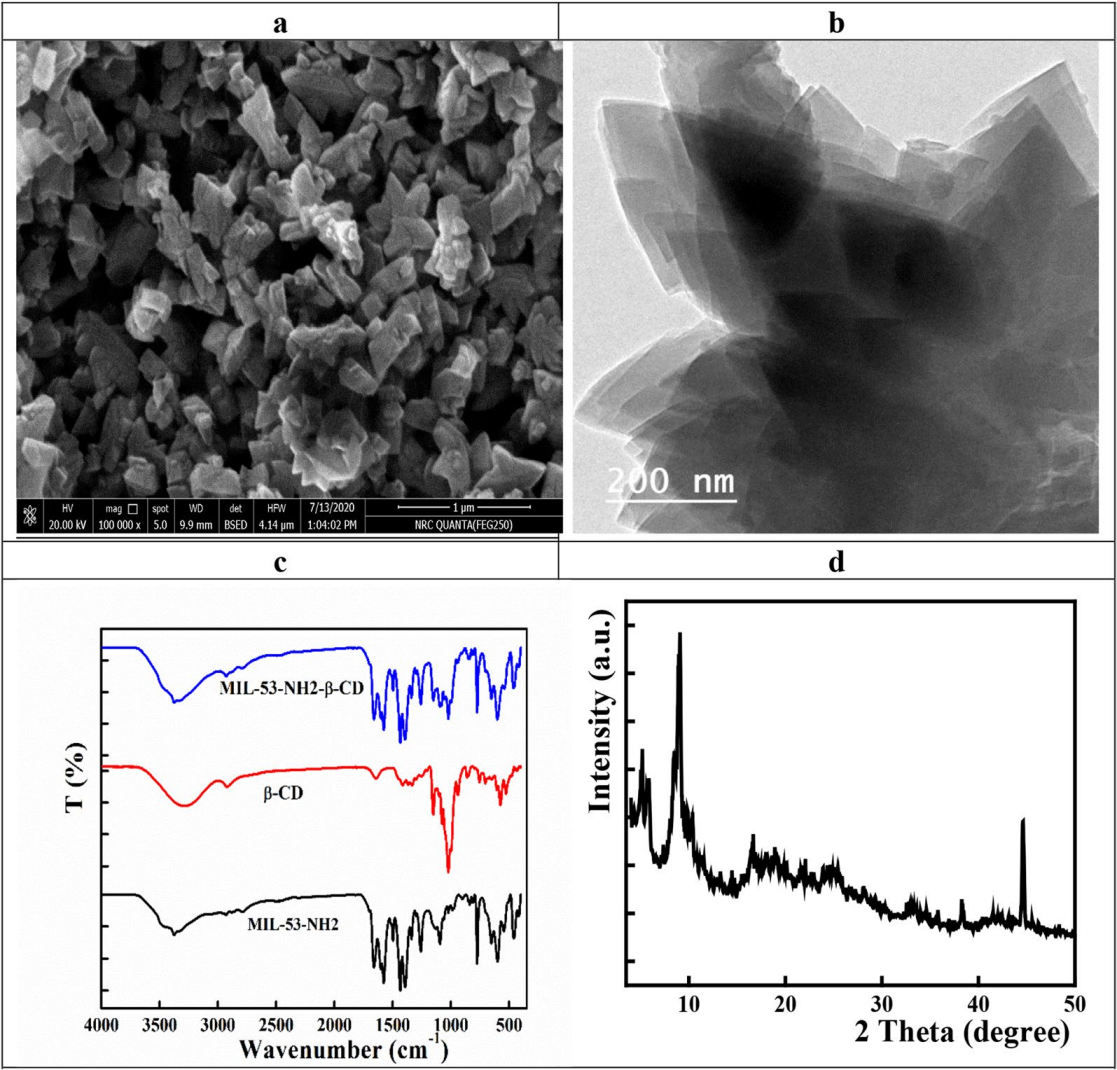
**a** SEM, **b** TEM, **c** FTIR, and **d** XRD of MIL-53-NH_2_-β-CD composite

SEM and TEM analysis described the morphological features of the synthesized samples. MIL-53-NH_2_-β-CD exhibited cubic long strip structure with average diameter of sample was 84 nm (Fig. 1a, b).

The FTIR spectra of MIL-53-NH_2_, β-CD, MIL-53-NH_2_-β-CD are presented in Fig. 1c. MIL-53-NH_2_ show the main characteristic bands of amino group, carboxylic group and benzene ring stretching at 3350, 1620–1505, and 1404 cm^−1^, respectively. β- CD showed characteristic bands known as saccharides at 1640 cm^−1^ (O–H bending vibration), 3400 cm^−1^ (O–H stretching vibration), 2930 cm^−1^ (C-H stretching vibration), and 1155 cm^−1^ (C-O vibration). α- Type glycosidic bond showed band located at 855 cm^−1^, which confirmed that CDs were consisted of glucopyranose units with α-1, 4-glycosidic bond. The peak at 3400 cm^−1^ was related to O–H stretching vibration.

FTIR spectrum of the cross-linked MIL-53-NH_2_-β-CD composite showed the main characteristic bands of both MIL-53-NH_2_ and β-CD. For confirming the composite (MIL-53-NH_2_-cross-linked β-CD*),* the changes in the intensity, shape and shift of the characteristic peaks can give considerable information about the chemical structure transformation. Peaks recorded for the cross-linked composite were wider than that of parent one, indicating hydroxypropyl groups incorporation broke the intramolecular hydrogen bond formed between C-2 and C-3. Moreover, the peak at 2960 cm^−1^ corresponds to the anti-symmetric vibration of methelene groups.

XRD spectra were used to examine the crystalline skeleton of the MIL-53-NH_2_ and MIL-53-NH_2_-β-CD composites. Figure 1d shows the MIL-53-NH_2_ and MIL-53-NH_2_-β-CD composite XRD diffraction patterns. The compounds exhibits XRD peaks at 2θ values of 9.1°, 12.4°, 17.5°, 24.5°, 25.9°, 38.1°, and 44.6°, as shown in Fig. 00, which validates the structure of MIL-53-NH_2_ [17]. The MIL-53-NH_2_ and MIL-53-NH_2_-β-CD composite's distinctive peaks are seen at the same angles, suggesting that MOF's crystalline structure does not change after β-CD impregnation. However, after modification with β-CD, the associated peaks widen, which is explained by the degree of crystallinity declining.

### Optimization of the sensing matrix

#### Nature of the sensing element

**Fig. 2:**
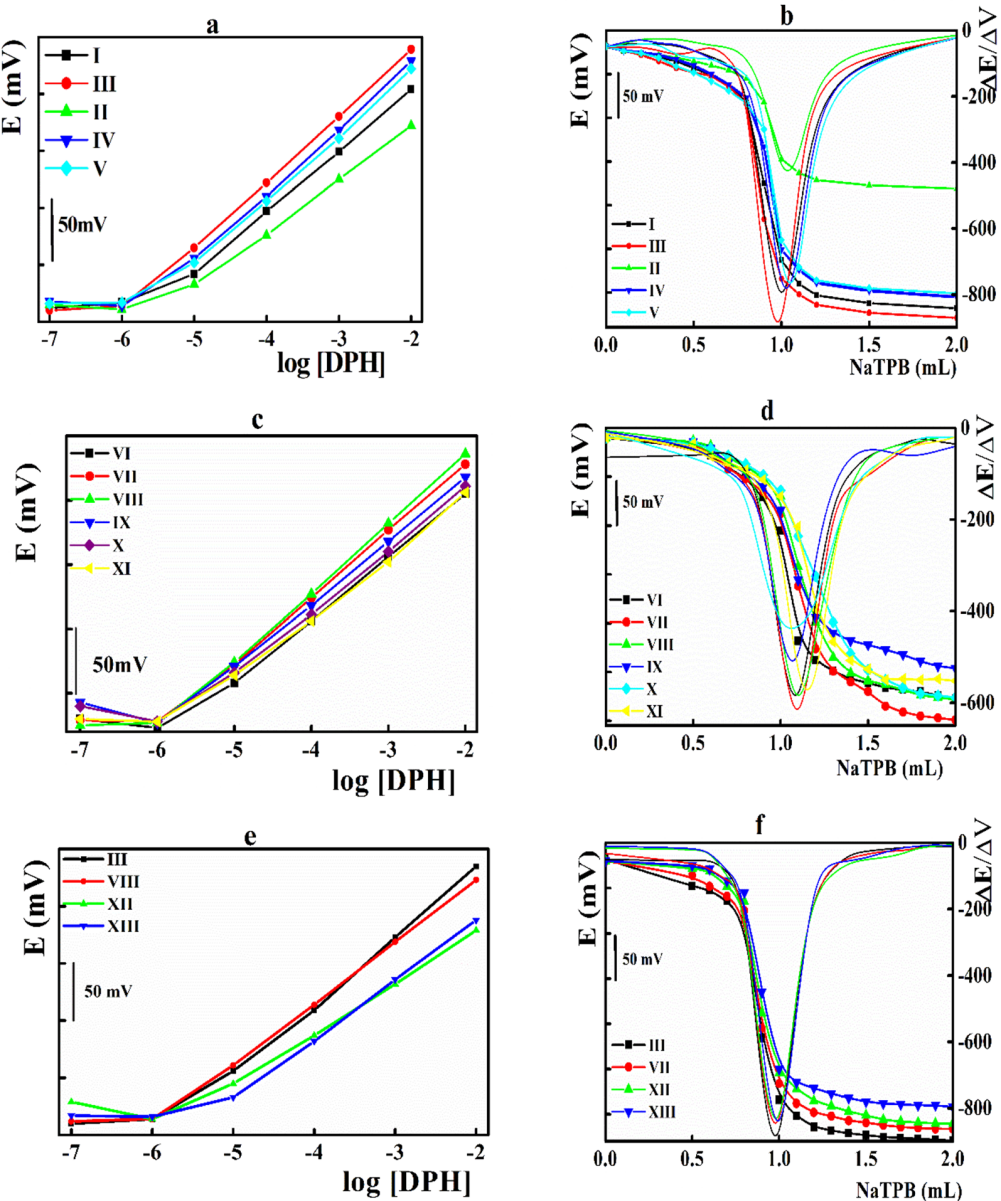
**a**, **c**, **e** Potentiometric behavior of the disposable sensors integrated with different sensing elements, and **b**, **d**, **f** potentiometric titration of DPH versus NaTPB solution applying the disposable sensors integrated with different sensing elements as indicator electrode

The electrode matrix was enriched with various macrocyclic compounds including cyclodextrin, crown ether, and calixarene derivatives **(I to XIII)**. The blank electrodes, containing NaTPB as ionic sites, exhibited sub-Nernstian response values (42.2 ± 2.2 mVdecade^−1^) within a narrow linear DPH concentration range. Upon fortification with molecular recognition elements, improved potentiometric responses were recorded based on the nature of the sensing element (Fig. 2).

Among various cyclodextrin derivatives (Fig. 2a), the native β-CD (**III**) exhibited the proper Nernstian response (60.4 ± 1.5 mVdecade^−1^) within the DPH concentration ranged from 10^−6^ to 10^−2^ molL^−1^. On the other hand, lower Nernstian responses (54.9 ± 2.7 and 47.8 ± 1.0 mVdecade^−1^) were reported for both α- and γ-CDs (**I, II**), respectively based their improper cavity size for fitting the DPH molecule. Side derivatization of the β-CD ring with the methyl groups (**IV, V**) showed slightly lower Nernstian response values with postulated steric hindrance. The lipophobic interior CD cavity offers a suitable environment for trapping the nonpolar part (aromatic benzene ring of the DPH molecule and formation of a stable inclusion complex. The stability constants of the formed inclusion complex are governed by the size of both CDs cavity, and the side substitution of the CD ring [50–53]. The postulated mechanism was illustrated in Figure S2.

Performing DPH potentiometric titration against NaTPB using the fabricated sensors as indicator electrodes, a similar trend was reported as β-CD (**III**) exhibited the highest total potential jump (311 mV) (Fig. 2b).

Crown ethers showed a conformation with a lipophobic cavity capable of fitting the guest molecule [50, 52]. The ring size relative to the gust molecule governed the stability constant of the formed CEs-DPH inclusion complex. In the present study, the listed crown ethers recorded Nernstian slope values based their cavity size (Fig. 2c). Among them, 15-crwon-5 ether (**VII**) and 18-crwon-6 ether (**VIII**) showed the highest Nernstian responses (52.1 ± 0.3 and 52.6 ± 0.8 mVdecade^−1^), compared to other crown ether derivatives with larger cavity size (47.8 ± 2.3 mV decade^−1^ for 30-crown-10 ether **XI**). The same conclusion was recommended under potentiometric titration **(**Fig. 2d**)** suggesting crown ether (**VII**) as sensing ionophore.

Calixarenes are cavity-shaped cyclic oligomers made up of phenol units linked via alkylidene groups that can form host–guest inclusion complexes with a large variety of organic drug molecules [62]. In the present study, the performance of the sensors integrated with the selected β-CD (**III**), and crown ether (**VIII**), were compared with those containing calix-4-arene (**XII**), and calix-8-arene (**XIII**). The results indicated the superiority β-CD **(III)** compared with the other used ionophores under either direct potentiometric or potentiometric titration measurements (Fig. 2e, f).

**Fig. 3:**
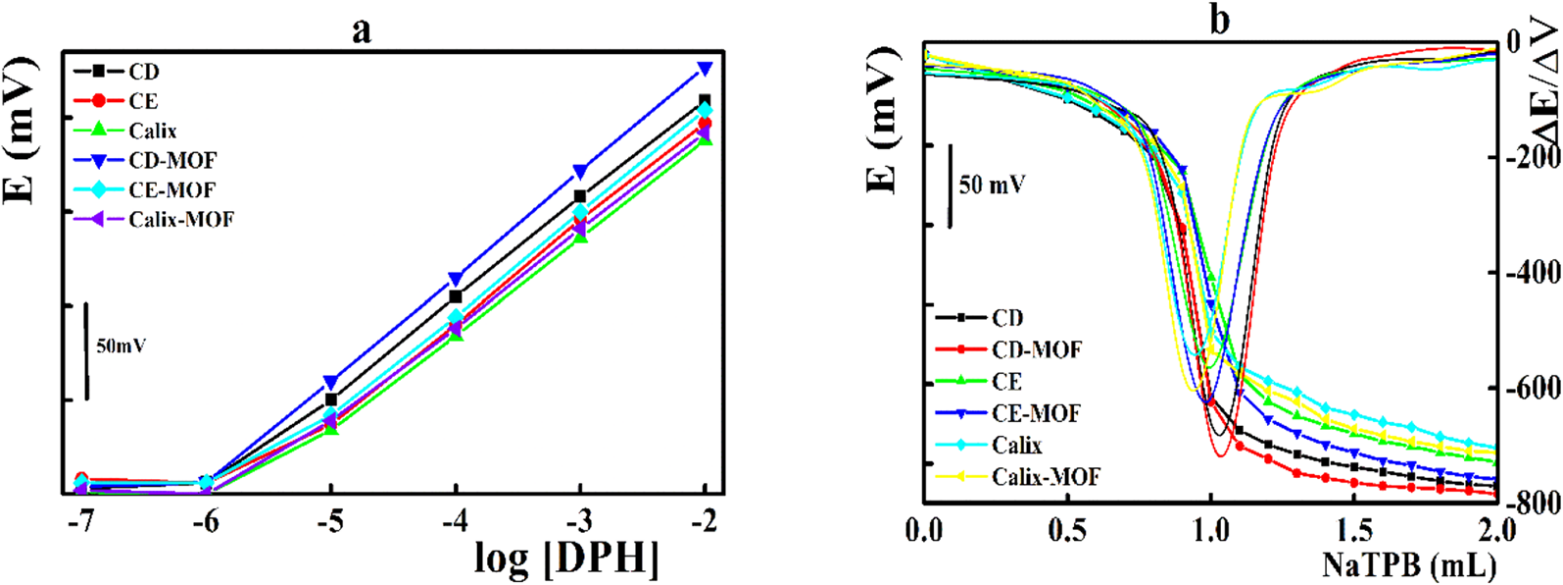
**a** Potentiometric behavior of the disposable sensors integrated with different sensing elements, **b** potentiometric titration of DPH versus NaTPB solution applying the disposable sensors integrated with different sensing elements as indicator electrodes

Next, the aforementioned selected sensing macromolecules were cress-linked with **MIL-53-NH**_**2**_, and the electrode matrix was enriched with the cross-linked compound instead of the free ionophore (Fig. 3). Noticeable enhancement of the electrode response upon cross-linking with the MOF was recorded based on the enhanced surface area and availability of more sensing sites. Integration of the electrode matrix with β-CD-MOF composite showed the best performance under direct potentiometric and potentiometric titration measurements.

**Fig. 4:**
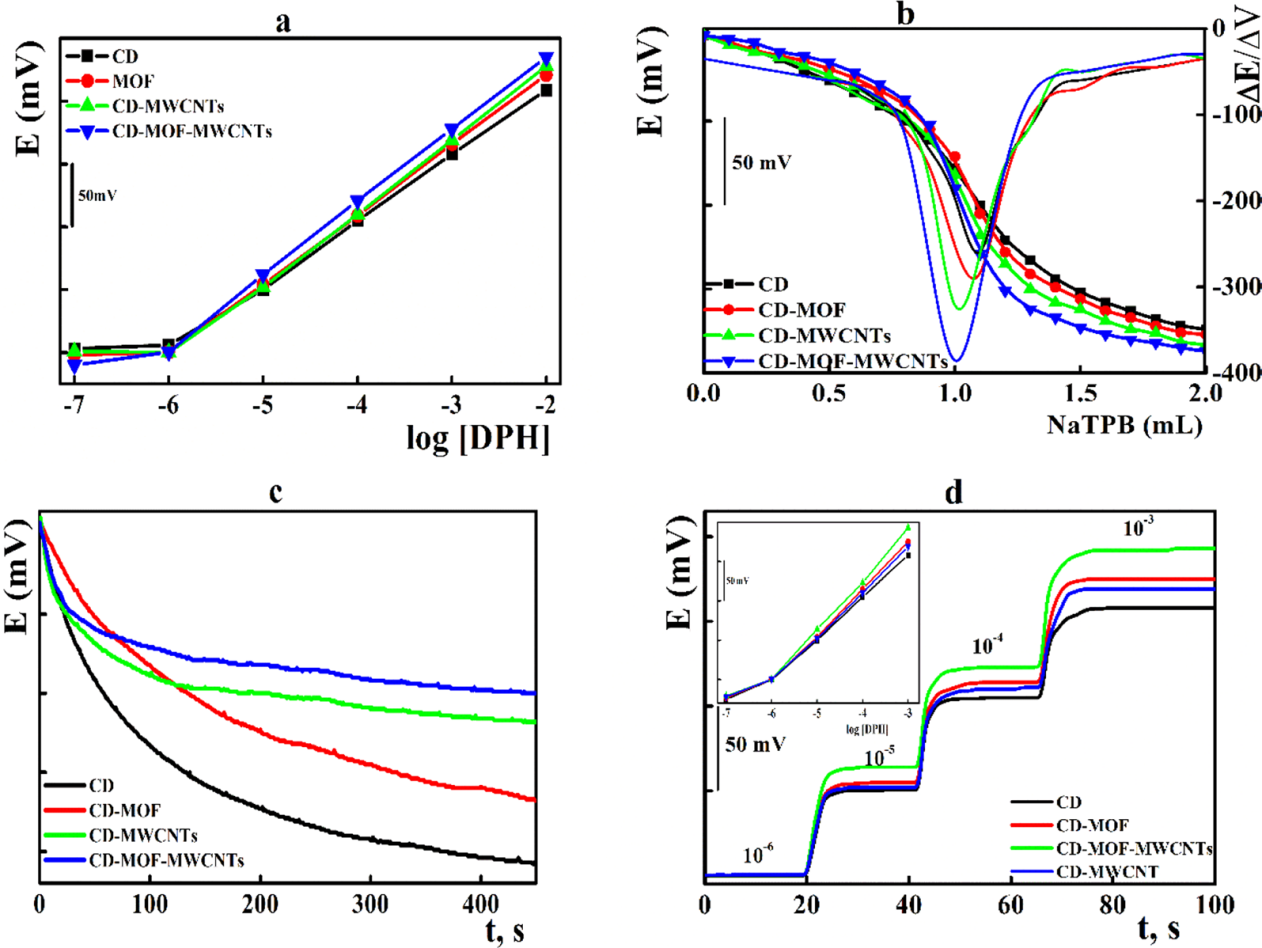
Influence of the sensing element on; **a** potentiometric response; **b** potentiometric titration of DPH versus NaTPB; **c** preconditioning time; **d** electrode response time

Nanomaterials, with their unique promising futures, enhance the electroactive surface area and the transduction of the possible chemical interaction to a measurable electrical signal within the electrode matrix, which improve the sensitivity of the sensor [1–6]. The potentiometric response of the printed sensors integrated with either the free β-CD or β-CD-MOF cross-linked composites were evaluated in the presence of different carbonaceous nanomaterials such as MWCNTs and SWCNTs. Incorporation of MWCNTs improved the sensors performance with the highest Nernstian compliance 58.5 ± 0.6 mV decade^−1^ and the total potential change under potentiometric titration mode (Fig. 4a, b).

Following the sensor fabrication protocol, the solid state nature required short preconditioning time (the time required to record stable potential reading about ± 1.0 mV for the fresh electrode) compared with the PVC and liquid membrane electrodes. Moreover, incorporation of nanomaterials within the electrode matrix improves the hydrophobicity of the membrane surface which stabilized the electrode potential reading. Thus, sensors integrated with MWCNTs achieved potential stability reading within very short preconditioning time (less than 5 min) compared with the corresponding carbon nanotube matrices (Fig. 4c).

For flow injection analysis and enzymatic reactions, the electrode response time (defined as the time required to attain 90% of the total potential jump after sudden tenfold increase in the analyte concentration) is usually questionable. The impact of the sensing element on the sensor response time was monitored within DPH concentration ranged from 1 × 10^–6^ to 1 × 10^−3^ molL^−1^ (Fig. 4d). The fabricated sensors showed fast response time (ranged from 3 to 10 s), and the β-CD-MOF-MWCNTs based sensors were the best.

Next, the electrode matrix was enriched with different β-CD-MOF-MWCNTs content ranged from 0.0 to 5.0 mg. Ascending Nernstian slope values from 42.2 ± 2.2 for the blank electrode to reach about 56.0 ± 0.3 mV decade^−1^ for 2.0 mg were recorded. Higher concentrations showed lower Nernstian slope values (52.3 ± 0.9 mV decade^−1^ for 5.0 mg) due to the over saturation of the membrane matrix which hinder the complex formation between DPH and CD moiety.

#### Anionic sites effect

Cyclodextrins (CDs) behave as neutral carrier ionophores, therefore, cyclodextrin based sensors response only in the presence of charged ionic sites [63–65]. The presence of such ionic sits minimizes the sensing membrane resistance, enhances the ion exchange kinetics at the sensing membrane surface, and attracts the target analyte to the membrane surface for inclusion complex formation which improve the modified sensor selectivity and sensitivity.

Herein, the DPH performances characteristics of the sensors integrated with different tetraphenylborate derivatives and β-CD-MOF-MWCNTs as a promising sensing element were illustrated in Figure S3a. Improved sensitivities were recorded compared to the blank electrode, and NaTFPB showed the highest response (Nernstian response were 30.8 ± 0.5, 54.8 ± 0.7, 60.6 ± 0.4, and 58.0 ± 1.1 mVdecade^−1^ for the bare, NaTPB, NaTFPB, and KTClPB, respectively). Potentiometric titration applying sensors fortified with different anionic sites sustained this concept as the highest potential jump was recorded in the presence of NaTFPB (Figure S3b).

#### Membrane plasticizer

The electroanalytical performance of the ionophore-based potentiometric sensors is mainly controlled by the membrane polarity, which is based on the dielectric constant value of the applied plasticizer. The membrane plasticizer governed the mobility of the sensing element and the formed inclusion complexes [65, 66]. For comparison, plasticizers having different dielectric constants were used including; *f*-PNPE, *o*-NPOE, TCP, DOP, and DOS (ɛ = 50, 24.8, 17.6, 3.8, and 5.2, respectively) [67]. Sensor fabricated using high polar plasticizers showed ideal Nernstian slope values (60.1 ± 0.4 and 60.7 ± 1.5 mVdecade^−1^ for *o*-PNPE and *f*-NPOE, respectively) (Figure S4a).

Potentiometric titration measurements showed total potential jump and inflection at the end point related to the dielectrical constants of the aforementioned plasticizers [58]. The potential jump values were 165, 135, 118, 182, and 188 mV for TCP, DOS, DOP, *o-*NPOE and *f-*PNPE, respectively (Figure S4b).

### Sensor performance

The performance characteristics of DPH sensors integrated with either free β-CD, β-CD-MOF or β-CD-MOF-MWCNTs as molecular recognition elements was evaluated following the IUPAC recommendation (Table 1) [56]. The β-CD-MOF-MWCNTs-based sensors showed the best performance with ideal Nernstian slope value of 60.7 ± 1.5 mV decade^−1^, and low detection limit 3 × 10^−7^ molL^−1^.

**Table 1:** Performance characteristics of various DPH screen-printed sensors

Parameter/sensing element	β-CD	β-CD-MOF	β-CD-MOF-MWCNTs
Linear range (molL^−1^)	10^−6^–10^−2^	10^−6^–10^−2^	10^−6^–10^−2^
Slope (mV decade^−1^)	52.9 ± 0.68	58.51 ± 1.2	60.7 ± 1.50
R	0.9992	0.9990	0.9994
LOD (molL^−1^)	1.0 × 10^−6^	7.0 × 10^7^	3.0 × 10^–7^
Response time (s)	10	8	3
Preconditioning time (min)	10	10	5
Shelf-life time (week)	4	12	24

Screen printing technique offers large-scale production of planner electrochemical sensors with high fabrication reproducibility. Herein, ten printed sensors showed average Nernstian slope value 59.7 ± 1.1 mVdecade^−1^. Due to the absence of the internal reference solution and the solid nature of the fabricated sensors, prolonged lifetime about 24 weeks was achieved during which stable Nernstian responses (± 2 mVdecade^−1^) was recorded. Moreover, the same fabricated disposable sensors can contentiously operate up to 4 weeks without loss of their performance.

The electroanalytical performance of the present DPH sensors was compared with the previously reported sensors [42–44]. The presented disposable sensors offer enhanced performance regarding their linear range, detection limit, fast response time, prolonged operational lifetime and the possibility of miniaturization and commercialization (Table S1). Moreover, as the introduced sensors are disposable, relatively short preconditioning time was required with the advantages of mass production and high fabrication reproducibility.

For appropriate application of a developed potentiometric sensors, the working pH range is a crucial operating issue. The impact of the pH value on the potential response was monitored in a wide pH value ranged from 2 to 9 (Figure S5). Stable and reproducible electrode potentials were recorded within the pH ranged from 2 to 6 which are near the biological samples. At lower pH values, the sensor responses are severely influenced by H_3_O^+^, while at higher pH values, the electrode potential dramatically decreased due to the formation of un protonated DPH species (pKa value for DPH is 8.84).

### Selectivity and degradation studies

The sensor selectivity towards the excipients and additives present in the marketed pharmaceutical formulations is a crucial issue. The selectivity of a novel analytical approach reflects its ability to detect the target analyte in the coexistence of excipients and other interfering species. The selectivity of the potentiometric sensors was used to differentiate interfering species about target analyte by selectivity coefficient [68]. Usually, matched potential method (MPM) was more suitable to estimate the selectivity coefficient for the neutral compounds or different charged analytes [69]. Herein, the selectivity of the β-CD-MOF-MWCNTs based sensors against donepezil was promoted (Table 2) via inclusion complex formation between DPH molecule and cyclodextrin moiety within the nanocomposite structure.

**Table 2:** Potentiometric selectivity coefficients-of the β-CD-MOF-MWCNTs integrated sensors

Interferent	-log* K*_*A,B*_	Interferent	-log* K*_*A,B*_
Li^+^	3.80	Maltose	3.90
NH_4_^+^	3.40	Starch	3.74
Ca^2+^	3.10	Sucrose	3.60
Mg^2+^	3.44	Glucose	3.29
Ni^2+^	3.90	Fructose	3.35
Co^2+^	3.53	Glycine	2.85
Phosphate	3.70	Caffeine	3.40
Citrate	2.54	Cysteine	2.80

^a^ Lower and upper concentrations of DPH, 10^–5^ and 10^–2^ molL^−1^ respectively

Drug additives and the degradation profiling of a pharmaceutical compound mean the studying and quantifying of the various degradants in bulk materials. Detection and monitoring of those contaminants is the most crucial issues in the modern pharmaceutical industry. For a newly introduced drug, the applied analytical techniques must be capable for monitoring of the parent active pharmaceutical compounds and degradants as recommended by ICH Guideline [59]. Moreover, some of the unidentified impurities and degradants formed during the shelf life may be health hazards, and therefore, identification and quantification of such impurities should be taken in consideration to show the safety of the final pharmaceutical product [70, 71].

The forced degradation studies were performed to provide adequate information to explore and identified the possible degradation pathways and formed degradants. Authentic donepezil material and commercial samples were exposed to forced and hydrolytic degradation studies [61]. Results indicated degradation of about 13% of DPH by refluxing in after 1 h an acidic medium, and 55% was degraded in the case of alkaline medium. On the other hand, oxidative stress degradation proceeded by degradation of 9% of the initial DPH with attacking of the electroactive amino group. Considering the thermal degradation and photolytic degradation of DPH, negligible degradation (< 2%) was recorded. None of these degradation products exhibited noticeable interference in potentiometric titration of DPH against NaTPB. The reported interference and degradation studies devoted the application of the potentiometric approach for determination of DPH in the pharmaceutical samples as indicating method.

### Electroanalytical applications

#### Potentiometric titration

**Fig. 5:**
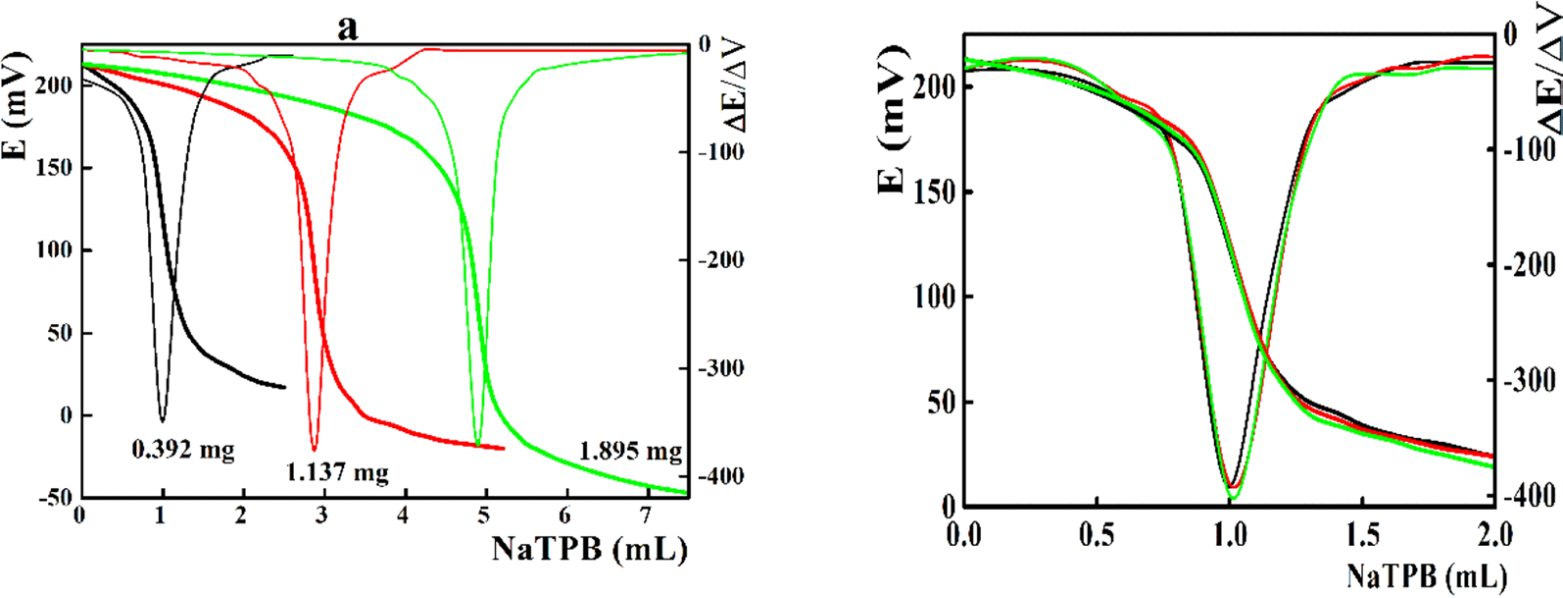
**a** Potentiometric titration of DPH against NaTPB using β-CD-MOF-MWCNTs based sensors, and **b** titration reproducibility for 0.392 mg DPH against NaTPB

Potentiometric titration is one of the most efficient electroanalytical potentiometric tools with high accuracy and precision than the direct potentiometric mode which require calibrations of the measuring cells [58]. For potentiometric titration of DPH against NaTPB, the presented disposable sensors integrated with β-CD-MOF-MWCNTs nanocomposite showed sharp and reproducible titration curves (ΔE values ranged from 196 to 264 mV) within DPH concentration ranged from 0.379 to 1.895 mg (Fig. 5 a). Moreover, high reproducibility with total average recovery of 100.5 ± 2.51% was recorded when titrating of 0.379 mg DPH (Fig. 5 b).

##### Flow injection analysis

**Fig. 6:**
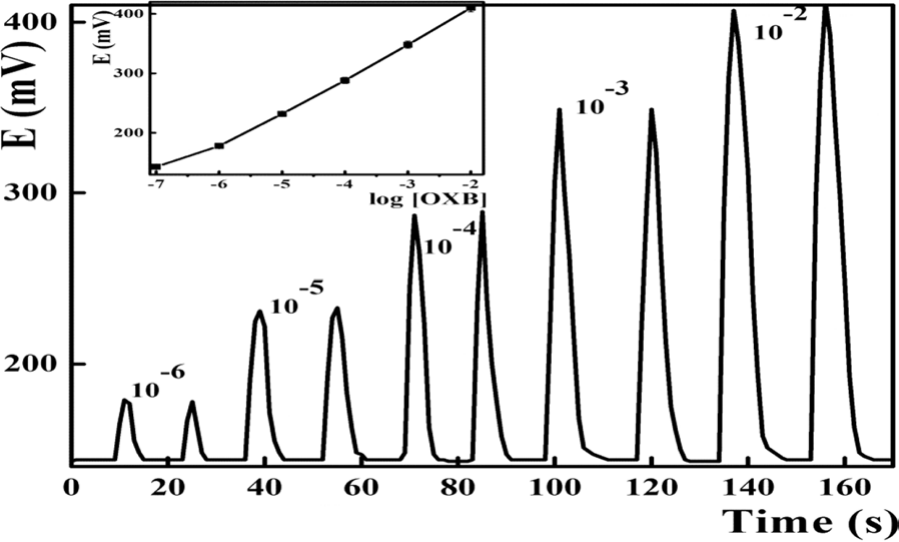
FIA potentiometric analysis of DPH with β-CD-MOF-MWCNTs based sensors

Due to its high sampling frequency, and improved reproducibility, potentiometric sensors were incorporated as a detector in flow injection systems [72]. Both the response time and the linearity range of the potentiometric sensor controlled its behavior in flow injection systems [73]. Herein, the modified DPH sensor showed stable potential readings which in turn decrease the FIA peak residence time and increased the sampling output up to 90 samples h^−1^. Calibration graphs (Fig. 6) were illustrated via injection of 50 µL DPH authentic solution in the carrier stream covering the DPH concentration range from 10^–6^ to 10^–2^ molL^−1^ with Nernstian response of 61.0 ± 1.1 mVdecade^−1^.

##### Sample analysis

Based on the recorded satisfactory improved sensitivity and selectivity, the modified sensors can be encouraged as a suitable tool for DPH analysis in various biological and pharmaceutical samples under direct potentiometric measurement, potentiometric titration and flow injection modes. As tabulated in Table 3, satisfactory agreement was achieved for DPH in various samples using the modified sensor and official methods (Table 3).

**Table 3:** Potentiometric determination of DPH in the marketed pharmaceutical formulations and biological samples

Sample	Taken (µg)	Aricept^®^			Spiked urine				Spiked Plasma		
		Found (µg)	Recovery	RSD	Found (µg)		Recovery	RSD	Found (µg)	Recovery	RSD
Standard addition	3.79	3.823	100.87	1.7		3.615	95.3	3.4	3.64	96.2	2.6
	37.9	37.83	95.23	1.2		36.55	96.4	2.0	35.5	93.88	3.3
	379	372.30	98.23	1.9		367.63	97.0	2.2	358	94.46	1.9
Titration	379	360.05	95.0	1.4		348.68	92.0	3.7	347	91.55	3.6
	1137	1108.6	97.5	2.0		1091.5	90.0	3.0	1074.5	94.5	3.4
	1895	1847.7	97.8	1.5		1828.7	96.5	1.8	1790.8	94.5	2.7
FIA	3.79	3.845	101.45	1.55							
	37.9	38.21	100.81	1.82							
	379	374.15	98.70	1.88							

^a^ Mean recovery and relative standard deviations of three determinations

##### Dissolution behavior of DPH pharmaceutical tablet

Studying the absorption of the pharmaceutical active ingredient after the oral administration represents a crucial factor to explore the drug pharmacokinetics behavior and the active material release from the drug formulations and its entrance through the gastro-intestinal tract. Moreover, the in vitro dissolution studies are usually relevant to show the in vivo performance and drug product bioavailability, and finally, observing novel formulations that might have slower or faster release rate.

Recently, electrochemical sensors have been employed for the in-line analysis of dissolution studies [74–76]. The present work described the dissolution profiles of donepezil in the commercial pharmaceutical sample where the DPH released in the dissolution medium was monitored using the developed DPH sensor and the UV-spectrophotometric measurements at 271 nm. Results illustrated in Figure S6 confirmed the agreement in recoveries recorded by both techniques (± 3.0%). As a result, releasing of DPH was reported to be rapid (90% amount of DPH was released within 15 min) and complete after 30 min.

## Conclusions

The present study introduces the construction and electroanalytical validation of novel donepezil printed potentiometric sensors based on β-cyclodextrin cross-linked metal organic framework-multiwall carbon nanotube composite as recognition element. The β-CD-MOF-MWCNTs based sensors exhibit Nernstian compliance value of 60.7 ± 1.5 mV decade^−1^ and fast response time about 3 s and long lifetime about 24 weeks within the DPH concentration ranged from 10^−6^ to 10^−2^ mol L^−1^. The proposed planner sensor can be introduced for routine analysis of DPH in various samples, and monitoring of the degradation process and dissolution profile. The modified sensors show improvement in electroanalytical characteristics compared with the previously reported DPH sensors regarding their sensitivity, response time, dissolution and degradation mentoring, FIA and potentiometric titration measurements.

## Supplementary Information

Supplementary material 1.

## Data Availability

No datasets were generated or analysed during the current study.

## Abbreviations

DPH: Donepezil hydrochloride
β-CD: β-Cyclodextrin
MOFs: Metal frame works
MWCNTs: Multi wall carbon nanotubes
SWCNTs: Single wall carbon nanotubes
FIA: Flow injection analysis
LOD: Limit of detection
PVC: Polyvinyl chloride
NaTPB: Sodium tetraphenylborate
NaTFPB: Sodium tetrakis (4-fluorophenyl) borate
KTCIPB: Potassium tetrakis (4-chlorophenyl) borate
DOS: Dioctylsebacate
DOP: Dioctylphthalate
TCP: Tricresylphosphate
*o*-NPOE: *o*-Nitro-phenyloctylether
*f*-PNPE: 2-Fluorophenyl-2-nitrophenyl ether
DMF: Dimethyl formamide
SEM: Scan electron microscopy
TEM: Transmission electron microscopy
XRD: X-ray diffraction
FTIR: Fourier transform infrared spectroscopy
