# Supplementary material for: β-cyclodextrin cross-linked metal organic frameworks as a new sensing candidate for donepezil hydrochloride potentiometric sensors
Source: BMC Chem. 2025 May 29;19(1):150. doi: 10.1186/s13065-025-01521-2 (PMC12121035; doi:10.1186/s13065-025-01521-2)
Supplement: Supplementary file 1 — Supplementary material 1. [file 13065_2025_1521_MOESM1_ESM.docx]

|  |
| --- |
| **Figure S1 Synthesis of β-cyclodextrin cross linked-metal organic frame work composite** |

| 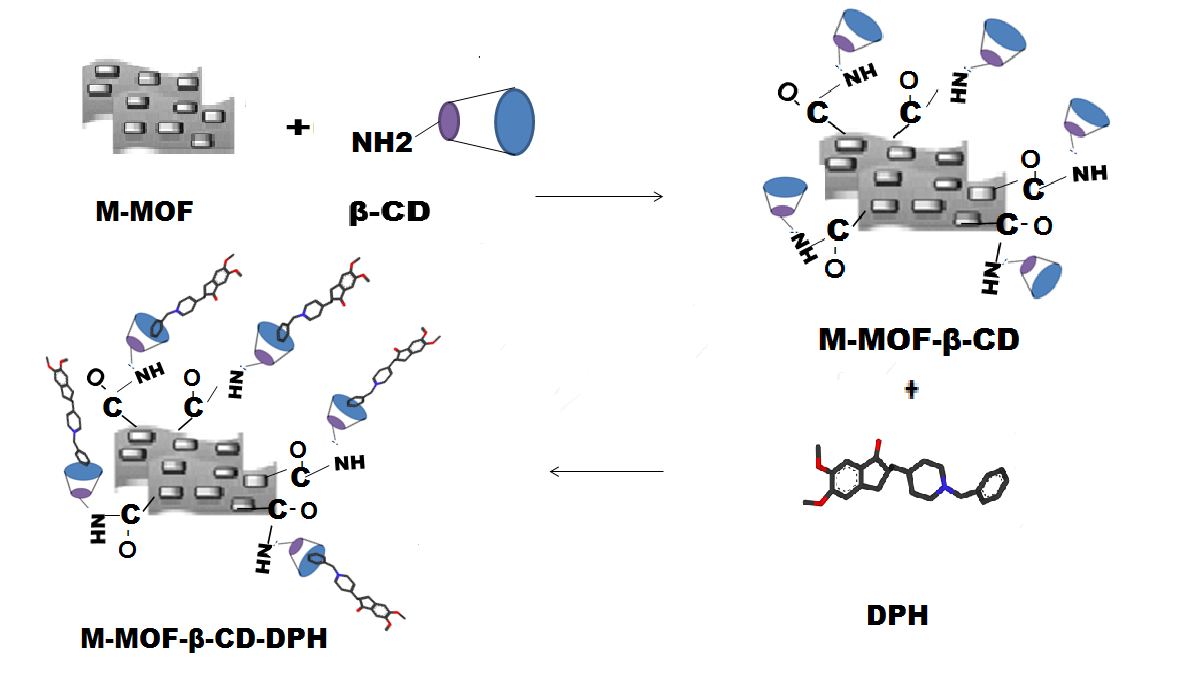 |
| --- |
| **Figure S2: Postulated mechanism for the synthesis of the cross-linked CD-MOF composite and the inclusion complex between CD and DPH molecule.** |

| **** | 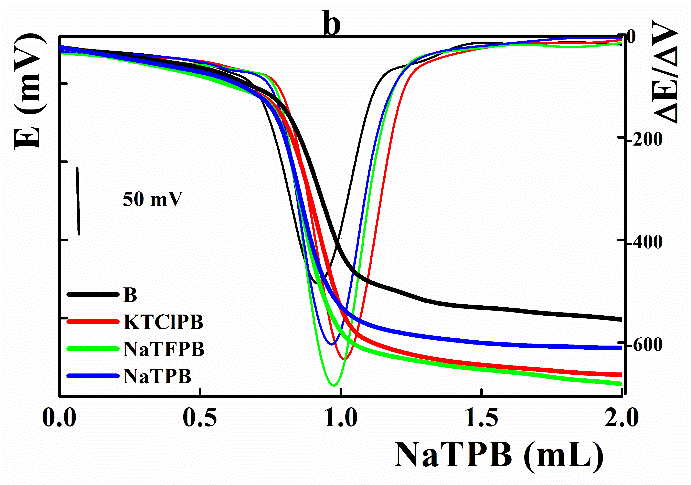 |
| --- | --- |
| **Figure S3 Impact of tetraphenylborate derivatives as ionic sites on: a) DPH sensor performance; b) DPH potentiometric titration against NaTPB solution.** | |

| **** | 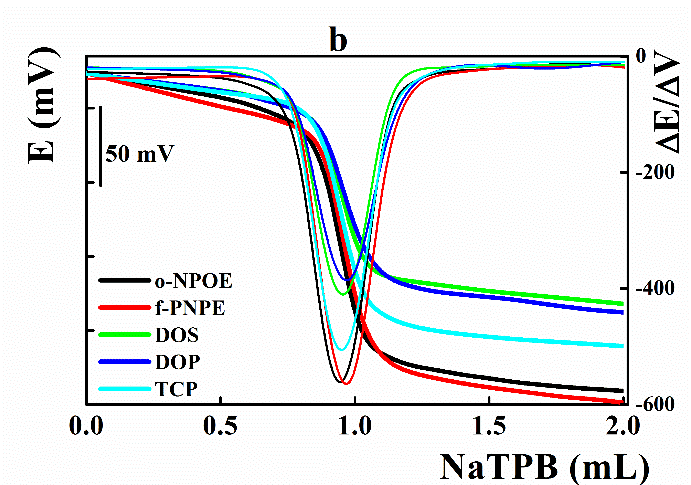 |
| --- | --- |
| **Figure S4 The membrane plasticizer impact on: a) DPH sensor performance; b) DPH potentiometric titration against NaTPB solution.** | |

|  |
| --- |
| **Figure S5 Performance of the fabricated DPH sensors at different working pH values** |

| **** |
| --- |
| **Figure S6 Monitoring of the dissolution profile for Aricept tablet (5 mg DPH/tablet) using DPH potentiometric sensor and UV-spectrophotometric measurements.** |

**Table S1: Comparison of the electroanalytical performances for different DPH sensors**

| **Sensors** | **Present** | **42** | **43** | **44** |
| --- | --- | --- | --- | --- |
| **Type of sensor** | **SPE** | **PVC** | **PVC** | **PVC, CPE** |
| **Electroactive material** | **β-CD-MOF-MWCNTs** | **DPH-ion pairs** | **DPH-ion pairs** | **DPH-TPB** |
| **Concentration range (molL^-1^)** | **10^-6^-10^-2^** | **10^-6^-10^-2^** | **10^-6^-8.0×10 ^-3^** | **10^-4^-10^-2^** |
| **Slope (mV decade^-1^)** | **60.7±1.5** | **54.0±0.5** | **58.51±0.8** | **57.0±0.4** |
| **DL (molL^-1^)** | **7.0×10^-7^** | **3.0×10^-6^** | **7.4×10^-7^** | **7.0×10^-7^** |
| **Titration range (mg)** | **0.379-1.895** | **3.79-11.37** | **------** | **20.8** |
| **Response time (s)** | **3** | **30** | **30** | **180-300** |
| **Preconditioning Time** | **5 min** | **24 h** | **2 h** | **1h** |
| **Shelf-life time (week)** | **24** | **4** | **6-8** | **8** |
| **Working pH range** | **2-6** | **4-8** | **5-8** | **3-7** |
| **Flow injection analysis** | **Done** |  |  |  |
